# The impact of Gam-COVID-Vac, an Adv5/Adv26 COVID-19 vaccine, on the biomarkers of endothelial function, coagulation and platelet activation

**DOI:** 10.1371/journal.pone.0293074

**Published:** 2023-10-18

**Authors:** Anar Turmukhambetova, Sergey Yegorov, Ilya Korshukov, Valentina Barkhanskaya, Svetlana Kolesnichenko, Dmitriy Klyuyev, Zhibek Zhumadilova, Aruzhan Pralieva, Laylim Absaghit, Ruslan Belyaev, Dmitriy Babenko, Gonzalo H. Hortelano, Matthew S. Miller, Dmitriy Vazenmiller, Irina Kadyrova

**Affiliations:** 1 Research Centre, Karaganda Medical University, Karaganda, Kazakhstan; 2 Michael G. DeGroote Institute for Infectious Disease Research, McMaster Immunology Research Centre, Department of Biochemistry and Biomedical Sciences, McMaster University, Hamilton, ON, Canada; 3 School of Sciences and Humanities, Nazarbayev University, Astana, Kazakhstan; 4 Department of Neurology, Psychiatry and Rehabilitology, Karaganda, Kazakhstan; King Saud University College of Medicine, SAUDI ARABIA

## Abstract

COVID-19 vaccines have played a critical role in controlling the COVID-19 pandemic. Although overall considered safe, COVID-19 vaccination has been associated with rare but severe thrombotic events, occurring mainly in the context of adenoviral vectored vaccines. A better understanding of mechanisms underlying vaccine-induced hypercoagulability and prothrombotic state is needed to improve vaccine safety profile. We assessed changes to the biomarkers of endothelial function (endothelin, ET-1), coagulation (thrombomodulin, THBD and plasminogen activator inhibitor, PAI) and platelet activation (platelet activating factor, PAF, and platelet factor 4 IgG antibody, PF4 IgG) within a three-week period after the first (prime) and second (boost) doses of Gam-Covid-Vac, an AdV5/AdV26-vectored COVID-19 vaccine. Blood plasma collected from vaccinees (n = 58) was assayed using ELISA assays. Participants were stratified by prior COVID-19 exposure based on their baseline SARS-CoV-2-specific serology results. We observed a significant post-prime increase in circulating ET-1, with levels sustained after the boost dose compared to baseline. ET-1 elevation following dose 2 was most pronounced in vaccinees without prior COVID-19 exposure. Prior COVID-19 was also associated with a mild increase in post-dose 1 PAI. Vaccination was associated with elevated ET-1 up to day 21 after the second vaccine dose, while no marked alterations to other biomarkers, including PF4 IgG, were seen. A role of persistent endothelial activation following COVID-19 vaccination warrants further investigation.

## Introduction

COVID-19 vaccination has occasionally been linked to rare yet severe venous and arterial clotting incidents, both with and without a decrease in platelet count [1, 2]. The majority of these thrombotic events have been related to commonly administered adenoviral vector vaccines, namely the ChAdOx1 CoV-19 vaccine AstraZeneca and the University of Oxford), and the Ad26.COV2.S vaccine (Janssen; Johnson & Johnson) [1, 2]. More recently, a 24-year-old woman experienced a fatal instance of vaccine-induced immune thrombocytopenia and thrombosis (VITT) after receiving the Gam-COVID-Vac ("Sputnik-V" by the Gamaleya Research Institute of Epidemiology and Microbiology) vaccine, showing symptoms on the seventh day after her vaccination [3].

Several haematological markers, including platelet number, d-dimer, fibrinogen, and platelet factor 4 (PF4)-specific immunoglobulin G have been associated with VITT syndrome. However, the precise processes leading to increased clotting tendencies after vaccination are still not entirely understood [1, 2]. Recent research, mainly from European studies on vaccine recipients from the general population, has noted alterations in clotting activation and endothelial function tied to the temporary inflammatory response in post-vaccination state [4–7]. It is essential to further investigate these changes, particularly in the context of vaccines like Gam-COVID-Vac, accounting for vaccination timing and prior natural exposure to SARS-CoV-2, for refining vaccine strategies and enhancing their safety and efficacy globally.

The Gam-COVID-Vac vaccine utilizes a recombinant adenovirus (rAd) vector approach, typically injected in two separate doses–the first with rAd26 and a booster, with rAd5, spaced 21 days apart. Starting from late 2020, this vaccine has been used in 71 countries spanning Latin America, Africa, and Asia, including Kazakhstan. Between February and September 2021, Kazakhstan’s COVID-19 vaccination initiative predominantly utilized Gam-COVID-Vac, with over 85% of vaccine recipients having been administered Sputnik-V [8]. During this extensive vaccination rollout, we embarked on a study to assess the safety, potential side effects, and immune reactions to Gam-COVID-Vac among a group with varied prior exposure to COVID-19 [9].

Following our earlier studies of “Sputnik-V” [9], here, we explored the effects of the vaccine on endothelial function, coagulation, and platelet activation and thus assessed changes to circulating endothelin (ET-1), thrombomodulin (THBD), plasminogen activator inhibitor (PAI), platelet activating factor (PAF) and PF4 IgG at three time points before and at 21 days following the prime and boost doses.

## Materials and methods

### Study setting

The current analysis is a follow-up to an earlier observational prospective study of safety and reactogenicity of Gam-COVID-Vac in Kazakhstan [9]. Venous blood samples were collected from all participants prior to first meal around a similar time in the morning. Due to a limited sample availability, only a subset of samples from the earlier study was analyzed. Briefly, the main study was conducted in Central Kazakhstan, uncovering the exposure to SARS-CoV-2 by spring 2021 [9–11]. Participants were recruited at the Karaganda Medical University in April-May 2021 (ClinicalTrials.gov #NCT04871841) and consisted of asymptomatic adults who had not previously received a COVID-19 vaccine; individuals with respiratory symptoms or a laboratory-confirmed COVID-19 diagnosis within two weeks prior to the study were excluded. Short questionnaires were administered to gather information on participants’ demographic background and recent history of COVID-19 exposure. At follow-up, participants were screened for respiratory symptoms and tested for COVID-19; participants with COVID-19 at follow-up were excluded. Gam-COVID-Vac administration (0.5 ml dose of vaccine injected into the deltoid muscle) followed the national guidelines and was conducted after sample collection [9]. The vaccine consisted of two doses: the first dose contained rAd26, and the second dose contained rAd5which were injected in 21 day interval according Gam-COVID-Vac injection protocol and National Ministry of Healthcare. Each dose contained 1± 0.5 x10^11^ rAd particles [9].

Based on the serologic spike (S) IgG and IgA findings in previous study [9], the "no prior COVID-19" group was defined by the absence of both S-IgG and S-IgA (IgG-, IgA-). The "prior COVID-19" group was identified by the positive level of either or both IgG and/or IgA (IgG+/-, IgA+/-) at the baseline [9].

### Ethics statement

All study procedures were approved by the Bioethics Committee of Karaganda Medical University under Protocol #12 (assigned number 45) from 06.04.2020. Written informed consent was obtained from all participants.

### Sample collection and processing

Three samples were taken: at the initial visit, and at day 21after dose 1 and -dose 2 Nasopharyngeal swabs (Med Ams, Russia) were obtained in accordance with the national COVID-19 testing protocols for following RT-PCR testing, to exclude participants with current SARS-CoV-2 infection. Additionally, 5 ml of blood was drawn using EDTA tubes (Improvacuter, Improve Medical Instruments,Guangzhou, China). Blood plasma was separated by centrifuging at 2,000×g for 10 minutes. Before further processing and analysis, all samples were aliquoted and stored at a temperature of -80°C. To mitigate the potential bias from varying runs, samples from all three stages (initial, post-first dose, and post-second dose) were tested on the same plate for biomarkers assay [9].

#### Biomarker assays

All ELISA assays were performed on blood plasma using commercially available ELISA kits (Cloud Clone Corp., China) for ET-1 (#CEA482Hu), PAI-1 (#SEA532Hu), TMBM (#SEA529Ca), PF4 IgG (#AEK505Hu) and PAF (#CEA526Ge) following the manufacturer protocol. Paired vaccinee samples were assayed on the same ELISA plate to avoid the effects of inter-plate variability. Absorbance was measured at OD450 nm using the Evolis 100 ELISA reader (Bio-Rad).

#### Statistical analyses

All analyses and graphing were performed in JASP 0.17.2.1 and Prism 9.5.1 software. We used the Wilcoxon Signed Rank Test to assess differences across the time points, while differences between the Prior and No Prior COVID-19 groups were assessed using Mann-Whitney U or Pearson’s Chi-squared tests. Since ET-1 and PAF assays use the competitive inhibition principle, the OD values for these biomarkers were inverted prior to analysis by subtraction from the highest measured OD within each assay. Other assay OD values were analyzed without any transformation.

## Results

We analyzed a total of 58 plasma samples paired across three study visits (baseline, post-dose 1 and post-dose 2) that were available from the original clinical trial.

Using the serological evidence of S-IgG and/or S-IgA, participants were classified based on their prior COVID-19 status as previously. Within the current study subset, there were no discernible demographic or biometric variations between the subgroups with or without prior COVID-19 exposure (Table 1). Due to the limited and variable sample availability, fewer samples were available for some of the assays, such as for PAF.

**Table 1:** Characteristics of study participants.

Characteristic	Overall, N = 58	No prior COVID-19, N = 23	Prior COVID-19, N = 35	p-value^a^
Age, years, median (IQR)	44.0 (37.3, 54.5)	44.0 (37.5, 53.5)	45.0 (38.0,54.0)	0.849
Male sex, n (%)	22 (37.9%)	16 (45.7%)	6 (26.1%)	0.132
Kazakh ethnicity, n (%)	35 (60.3%)	12 (52.2%)	23 (65.7%)	0.302
BMI, kg/m^2^, median (IQR)	25.1 (22.8, 27.6)	23.7 (22.3, 27.5)	25.3 (23.9, 27.7)	0.210
Any comorbidities ^b^	30 (51.7%)	12 (52.2%)	18 (51.4%)	0.665

^a^ Differences between the Prior and No Prior COVID-19 groups were assessed using Mann-Whitney U or Pearson’s Chi-squared tests.

^b^ Comorbidities consisted of self-reported gastrointestinal conditions, hypertension, chronic heart disease, chronic obstructive pulmonary disease, history of malignancy, diabetes, liver disease, thyroid dysfunction, kidney disease, neurologic conditions, autoimmune conditions; the distribution of individual comorbidities did not differ between the “no prior COVID-19” and “prior COVID-19” groups.

**Fig 1:**
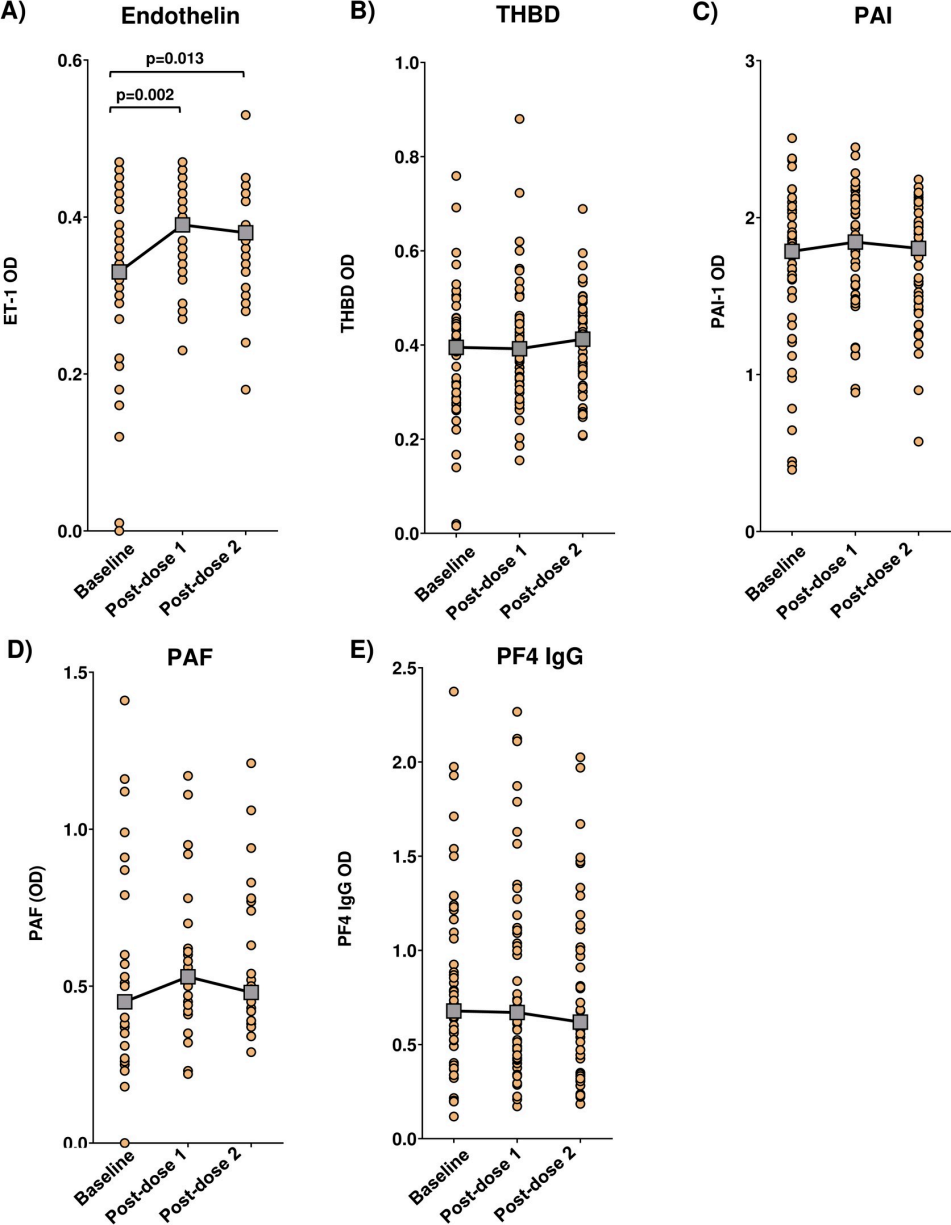
Impact of GamCovidVac vaccination on the blood biomarkers in all participants. A) Endothelin, ET-1 (n = 45). B) Thrombomodulin, THBD (n = 51). C) Plasminogen activator inhibitor, PAI (n = 49). D) Platelet activating factor, PAF (n = 27). E) Platelet factor 4 IgG antibody, PF4 IgG (n = 50). Each dot denotes a study participant, dashed lines link samples paired across the study visits. Gray boxes denote median OD at each visit. OD: optic density. Statistical significance was assessed by the Wilcoxon Signed Rank Test.

In the main analysis (Fig 1), unstratified by prior COVID-19 exposure, ET-1 was the only marker significantly impacted by vaccination. ET-1 was significantly increased post-dose 1 (p = 0.002), and this increase was sustained post-dose 2 compared to baseline (p = 0.013).

**Fig 2:**
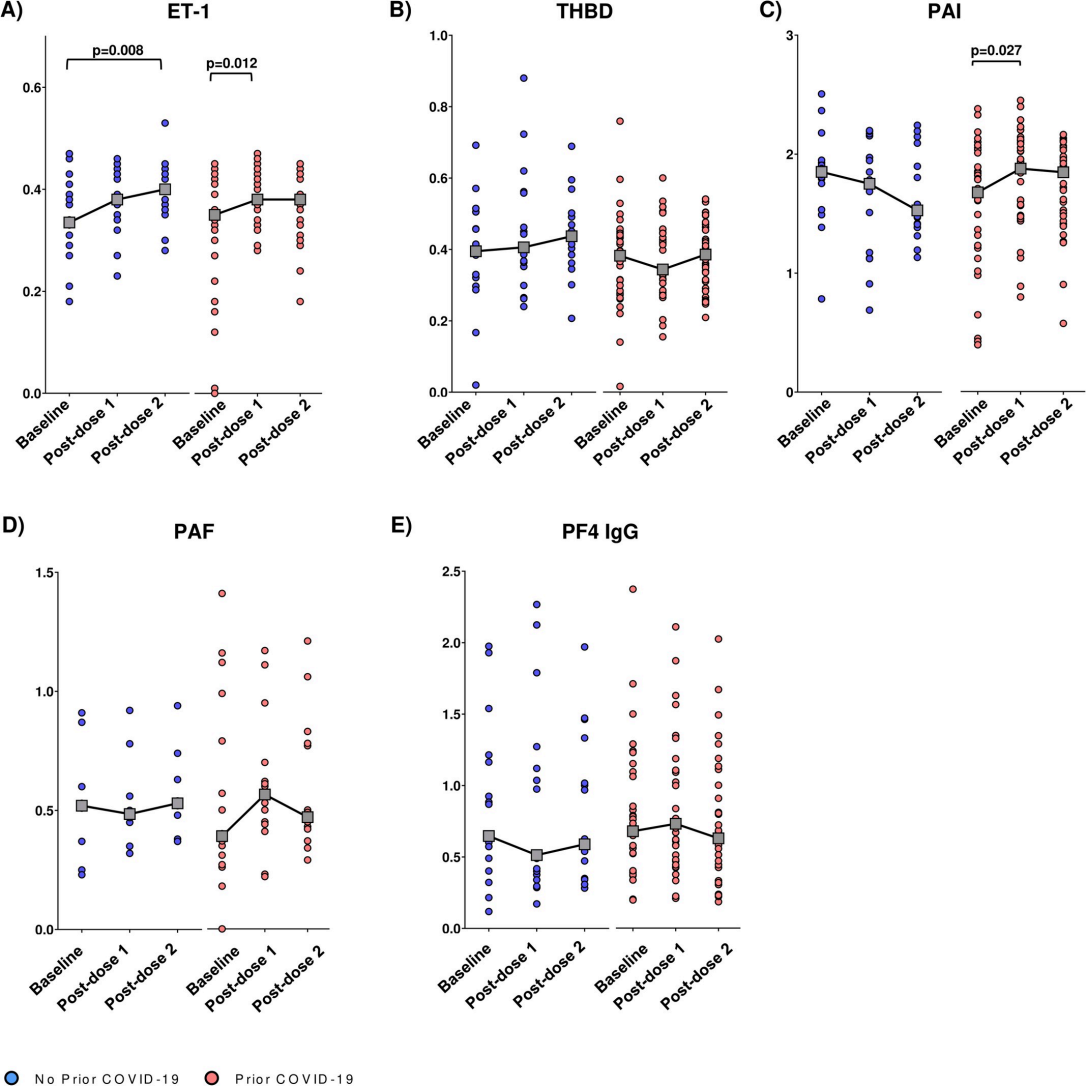
Impact of GamCovidVac vaccination on the blood biomarkers stratified by prior exposure to COVID-19. A) Endothelin, ET-1 (n = 16 and 29). B) Thrombomodulin, THBD (n = 17 and 34). C) Plasminogen activator inhibitor, PAI (n = 16 and 33). D) Platelet activating factor, PAF (n = 8 and 9). E) Platelet factor 4 IgG antibody, PF4 IgG (n = 17 and 33). Each dot denotes a study participant, dashed lines link samples paired across the study visits. Gray boxes denote median OD at each visit. OD: optic density. Statistical significance was assessed by the Wilcoxon Signed Rank Test. Lastly, a mild post-dose 1 increase (p = 0.027) was observed for PAI only in the prior COVID-19 group.

In the analysis stratified by prior COVID-19 exposure (Fig 2), ET-1 was elevated post-vaccination in both prior and no prior COVID-19 groups, although this increase was not sustained in the prior COVID-19 group post-dose 2.Lastly, a mild post-dose 1 increase (p = 0.027) was observed for PAI only in the prior COVID-19 group.

## Discussion

Here we studied the impact of Gam-COVID-Vac on endothelial function, coagulation, and platelet activation biomarkers within a three-week period after the first and second vaccine doses. Vaccination significantly increased ET-1 levels after the first dose, and this elevation was sustained after the second dose. Furthermore, post-dose 2 ET-1 elevation was most pronounced in vaccinees without prior COVID-19 exposure. Prior COVID-19 was also associated with a mild increase in post-dose 1 PAI. Consistent with earlier studies [4, 12], no marked alterations to other biomarkers, including PF4 IgG, were seen.

To the best of our knowledge, our study is the first to assess thrombosis-associated biomarkers in the context of Gam-COVID-Vac vaccination. The current lack of studies on this aspect of "Sputnik-V" is partly explained by a shortage of data on the vaccine’s safety and performance outside of Russia, where the vaccine originated [13]. At the same time, the reported cases of VITT and myocarditis after the receipt of Gam-Covid-Vac have raised concerns about the under-reporting of vaccine-associated vascular events [3, 14]. Our current findings support the data from both human and animal studies [5, 13, 15] that replication-deficient adenovirus vectored vaccines can trigger endothelial activation, which could potentially induce platelet aggregation and thrombosis.

ET-1, a potent vasoconstrictor produced by the vascular endothelium, is a key regulator of vascular tone and has been implicated in several cardiovascular diseases and in the pathogenesis of severe COVID-19 [16, 17]. An increase in ET-1 level may therefore suggest a disturbance in endothelial function following vaccination. In support of this, studies of ChAdOx-1 have found elevated post-vaccination levels of Von Willebrand Factor, another biomarker of endothelial function [5].

Interestingly, when stratifying the data by prior COVID-19 exposure, we noticed a mild increase in PAI levels in the prior COVID-19 group following the first vaccine dose. PAI is a primary inhibitor of fibrinolysis and higher levels may hint towards an increased coagulation state, which is also observed in COVID-19 [2]. This finding suggests that individuals with prior COVID-19 exposure might exhibit a more pronounced coagulation response after the first vaccine dose, possibly due to a more robust immune response triggered by the recognition of the SARS-CoV-2 S protein in the vaccine.

Since our research is a clinical trial, we monitored the clinical outcomes of the Gam-COVID-Vac vaccination. There was no identification of VITT syndrome or thromboses of other etiologies in participants of our study. However, three participants reported about exhibited exacerbations of chronic diseases such as psoriasis and arthritis. At the same time those individuals demonstrated the highest anti-PF4 levels.

Our study, while providing initial insights into the effects of the Gam-Covid-Vac was conducted on a small sample of 58 adults aged 44.0 (37.3, 54.5), recruited in Karaganda Medical University. In the context of the resources and samples accessible from the previous clinical trial, this cohort was selected for studies on biomarkers pertinent to endothelial function, coagulation, and platelet activation in relation to the Gam-COVID-Vac vaccination, based on the availability of funding and the anticipated feasibility [11]. We examined potential confounding factors such as gender, age, body mass index (BMI), and comorbidities (Table 1). Nevertheless, considering that the disparities between the groups were not statistically significant, these factors probably do not have a meaningful impact on the results. Our findings faced certain constraints in terms of generalizability, including age demographics, ethnic and genetic variation, health conditions, and geographic differences. The observed alterations noticed in ET-1 and PAI, though statistically notable, were slight, and their clinical importance remains unclear. To validate these observations and clarify their clinical significance, larger and more diverse studies are essential.

## Conclusion

Despite its limitations, our study provides preliminary yet crucial observations regarding alterations in biomarkers related to endothelial function, coagulation, and platelet activation associated with the Gam-COVID-Vac vaccination. The efficacy of COVID-19 vaccines in mitigating the pandemic’s spread is incontrovertibly significant. Nonetheless, the association of these vaccines with a rare but severe prothrombotic state necessitates further comprehensive research, particularly concerning the ramifications of sustained endothelial activation induced by the vaccine.

## Supporting information

S1 Data (CSV)
